# PD-1 inhibition disrupts collagen homeostasis and aggravates cardiac dysfunction through endothelial-fibroblast crosstalk and EndMT

**DOI:** 10.3389/fphar.2025.1549487

**Published:** 2025-03-17

**Authors:** Zejin Zhang, Zhenzhen Yan, Tao Yuan, Xiaona Zhao, Minghui Wang, Guoqing Liu, Lijun Gan, Wei Qin

**Affiliations:** ^1^ School of Pharmacy, Binzhou Medical University, Yantai, Shandong, China; ^2^ School of Pharmacy, Jining Medical University, Rizhao, Shandong, China; ^3^ School of Pharmacy, Shandong University of Traditional Chinese medicine, Jinan, Shandong, China; ^4^ School of Pharmacy, Shandong First Medical University, Jinan, Shandong, China; ^5^ School of Pharmacy, Shandong Second Medical University, Weifang, Shandong, China; ^6^ Department of Cardiology (Shandong Provincial Key Laboratory for Cardiovascular Disease Diagnosis and Treatment), Affiliated Hospital of Jining Medical University, Jining, Shandong, China; ^7^ The Key Laboratory of Cardiovascular Remodeling and Function Research, Chinese Ministry of Education and Chinese Ministry of Public Health, Qilu Hospital, Shandong University, Jinan, Shandong, China

**Keywords:** cardio-oncology, cardiotoxicity, PD-1 inhibitor, collagen distribution, EndMT

## Abstract

**Introduction:**

Cardiac immune-related adverse events (irAEs) from PD-1-targeting immune check-point inhibitors (ICIs) are an increasing concern due to their high mortality rate. Collagen plays a crucial role in maintaining cardiac structure, elasticity, and signal transduction; however, the effects and mechanisms of PD-1 inhibitor on cardiac collagen remodeling remain poorly understood.

**Methods:**

C57BL/6 mice were injected with anti-mouse PD-1 antibody to create a PD-1 inhibitor-treated model. Cardiac function was measured by echocardiography, and collagen distribution was analyzed with Masson’s trichrome staining and Sirius Red staining. Single-nucleus RNA sequencing was performed to examine the effects of PD-1 inhibition on gene expression in cardiac fibroblasts (CFs) and endothelial cells (ECs). EC-CF crosstalk was assessed using co-culture experiments and ELISA. ChIP assay was performed to analyze the regulation of TCF12 on TGF-β1 promoter. Western blot, qRT-PCR, and immunofluorescence staining were used to detect the expression of TCF12, TGF-β1, and endothelial-to-mesenchymal transition (EndMT) markers. Reactive oxygen species (ROS) levels were evaluated by DHE staining, MDA content, and SOD activity assays.

**Results:**

We report a newly discovered cardiotoxic effect of PD-1 inhibitor, which causes aberrant collagen distribution in the heart, marked by a decrease in interstitial collagen and an increase in perivascular collagen deposition. Mechanistically, PD-1 inhibitor does not directly affect CFs but instead impact them through EC-CF crosstalk. PD-1 inhibitor reduces TGF-β1 secretion in ECs by downregulating TCF12, which we identify as a transcriptional promoter of TGF-β1. This subsequently decreases CF activity, leading to reduced interstitial collagen deposition. Additionally, PD-1 inhibitor induces EndMT, increasing perivascular collagen deposition. The endothelial dysfunction induced by PD-1 inhibitor results from ROS accumulation in ECs. Inhibiting ROS with N-acetylcysteine (NAC) preserves normal collagen distribution and cardiac function in PD-1 inhibitor-treated mice by reversing TCF12 downregulation and EndMT in ECs.

**Conclusion:**

Our results suggest that PD-1 inhibitor causes ROS accumulation in cardiac ECs, leading to imbalanced collagen distribution (decrease in interstitial collagen and increase in perivascular collagen) in the heart by modulating TCF12/TGF-β1-mediated EC-CF crosstalk and EndMT. NAC supplementation could be an effective clinical strategy to mitigate PD-1 inhibitor-induced imbalanced collagen distribution and cardiac dysfunction.

## Graphical Abstract

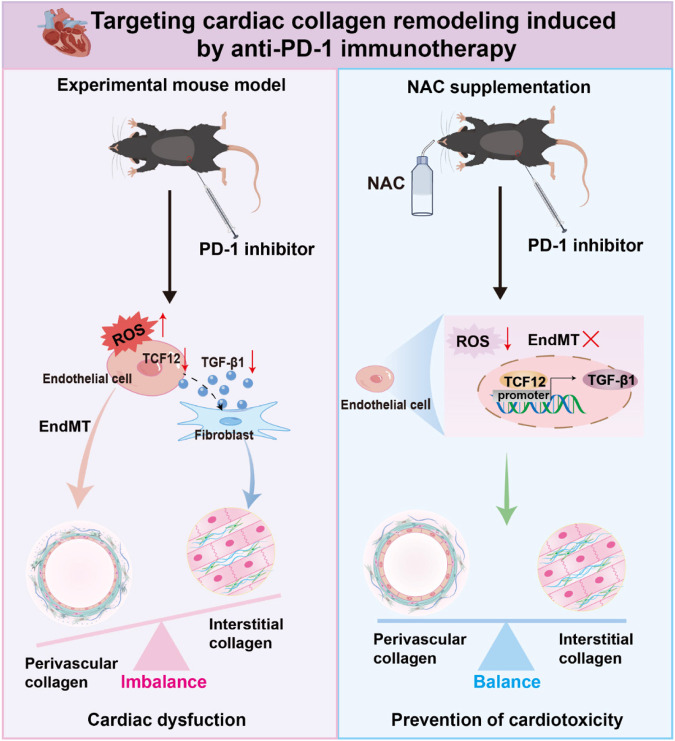

## 1 Introduction

Immune checkpoint inhibitors (ICIs) represent a significant advancement in cancer treatment and have been widely used in tumor immunotherapy. Programmed cell death-1 (PD-1) is a pivotal immune checkpoint in tumors. PD-1 inhibitors can block the interaction between PD-1 and PD-L1, thereby awakening immune-related cells and enhancing their ability to recognize tumor cells (Lin et al., 2023). However, studies have found the usage of PD-1 inhibitors may cause cardiotoxicity, such as myocarditis, cardiomyopathy, myocardial fibrosis, heart failure, and pericardial disease. Although uncommon, their occurrence can be sudden and fatal (Moslehi et al., 2021; Safi et al., 2021). Therefore, investigating early cardiac pathological changes and preventing detrimental effects induced by PD-1 inhibitors before the manifestation of severe complications is extremely important.

Collagen is the primary component of the cardiac matrix and is crucial for maintaining cardiac morphology, elasticity, and signal transduction. In clinical cases of cardiotoxicity induced by PD-1 inhibitors, many patients exhibit abnormal cardiac collagen deposition in their cardiac tissues, and the deposition may manifest independently of myocarditis (Heinzerling et al., 2016), suggesting that PD-1 inhibitor-induced cardiac collagen remodeling is not exclusively secondary to inflammation. Current research on PD-1 inhibitor-induced cardiotoxicity primarily centers on myocarditis mechanisms, with limited exploration into the mechanisms underlying PD-1 inhibitor-induced cardiac collagen remodeling. Therefore, investigating the effects and regulatory mechanisms of PD-1 inhibitors on cardiac collagen remodeling is essential for a comprehensive understanding of PD-1 inhibition-related cardiac adverse events.

Interspersed among cardiomyocytes (CMs), cardiac fibroblasts (CFs) primarily function to provide structural support during the thickening of the ventricular wall from embryogenesis through to adulthood (Ottaviano and Yee, 2011). The function of CFs can be regulated by autocrine signals or by signals from other cardiac cells through cell-to-cell crosstalk. For example, CMs are the primary functional cells of the heart, responsible for generating the contractile force that drives blood through the heart and the entire body. Studies have shown that CM-CF crosstalk is critical in regulation of collagen deposition, in which, CMs may promote fibrosis via miRNAs-containing exosomes (Tang et al., 2023). Endothelial cells (ECs) are one of the most abundant non-CMs in the heart and EC-derived miRNAs can also promote cardiac remodeling through the dynamic interaction between ECs and CFs (Wang et al., 2019). In addition to their communication with CFs, ECs can also contribute to collagen deposition through a process known as endothelial-to-mesenchymal transition (EndMT). EndMT is a complex biological process in which ECs lose their endothelial characteristics and acquire mesenchymal cell properties (Zhu et al., 2023). Under certain physiological or pathological conditions, ECs undergo EndMT to transform into CF-like cells, secreting collagen and promoting fibrosis (Cheng et al., 2021).

Transcription factor 12 (TCF12, also known as HTF4 or HEB) is a member of helix-loop-helix (HLH) protein family. It plays a crucial role in the cell development and differentiation of various tissues including skeletal muscle, neurons, mesenchymal tissue, and lymphocytes (Di Rocco et al., 1997; Li et al., 2017; Yi et al., 2017; Zhang et al., 1991). TCF12 has been identified as an oncogenic risk factor. Studies have shown that TCF12 promotes cancer metastasis in hepatocellular carcinoma, colorectal cancer, and melanoma (Lee et al., 2012; Tian et al., 2023; Yang et al., 2019). Additionally, TCF12 has been shown to induce angiogenesis in osteosarcoma (Li W. et al., 2024). In addition to cancer, TCF12 is involved in the regulation of other diseases. For instance, research has found that TCF12 accelerates the progression of osteoarthritis by targeting CXCR4 (Zheng et al., 2024). However, to date, there has been no research specifically investigating the role of TCF12 in cardiac function.

Oxidative phosphorylation is the primary method for generating energy in cells and is crucial for maintaining normal cellular function (Nolfi-Donegan et al., 2020). However, excessive activation of oxidative phosphorylation can lead to an increase in reactive oxygen species (ROS). While ROS play an important role in normal physiological processes, excessive ROS production that exceeds the body’s scavenging capabilities can result in oxidative stress within cells (Apel and Hirt, 2004). Oxidative stress is implicated in the etiology of many illnesses via causing the damage of diverse biological molecules. Previous studies have shown that excessive oxidative stress can lead to endothelial dysfunction (Giordo et al., 2021; Sobierajska et al., 2022). The tripeptide glutathione (GSH), which contains a sulfhydryl group, is synthesized and maintained at high levels in all cells, serving as a crucial defense mechanism against oxidative stress. N-acetylcysteine (NAC), a synthetic derivative of the naturally occurring amino acid L-cysteine, acts as a precursor to GSH and has antioxidant properties through replenishing intracellular levels of GSH. As a medication and dietary supplement, NAC has a variety of medical applications (Li X. et al., 2024; Raghu et al., 2021).

In this study, we investigated the impact of PD-1 inhibitor on cardiac collagen remodeling and utilized single-nucleus RNA sequencing (snRNA-seq) to characterize the transcriptomic profiles of individual cells in mouse hearts under normal conditions and following PD-1 inhibitor treatment. We specifically examined the effects of PD-1 inhibitor on ECs, CFs, and EC-CF communication. Our findings reveal a newly discovered cardiotoxic effect of PD-1 inhibitor, that PD-1 inhibition induces aberrant collagen distribution in the heart, characterized by reduced interstitial collagen and increased perivascular collagen deposition. Mechanistically, PD-1 inhibitor does not directly affect CFs but instead impact them through EC-CF crosstalk. PD-1 inhibitor reduces the expression of TCF12, which transcriptionally decreases the expression and secretion of TGF-β1 by ECs, subsequently resulting in reduced CF activity and interstitial collagen deposition through EC-CF crosstalk. PD-1 inhibitor also induces EndMT to increase perivascular collagen deposition. Overactivation of oxidative phosphorylation and ROS accumulation are responsible for the PD-1 inhibitor-induced endothelial dysfunction. Ultimately, we discovered that supplementing with NAC can reverse the aberrant collagen distribution and preserve cardiac function induced by PD-1 inhibitor. Our findings indicate that NAC supplementation may be a promising therapeutic avenue to prevent cardiotoxicity in the anti-PD-1 therapy.

## 2 Materials and methods

### 2.1 Treatment using a PD-1 inhibitor *in vivo*

Eight-week-old male C57BL/6 mice were purchased from Jinan Pengyue Laboratory Animal Breeding Co., Ltd (Jinan, China) and randomly assigned to two groups: Control group and PD-1 inhibitor group. For the groups treated with the PD-1 inhibitor, mice were intraperitoneally injected with InVivoPlus anti-mouse PD-1 antibody (Bioxcell, West Lebanon, United States) at a dose of 5 mg/kg on the 1st and 14th days of a 28-day cycle. To verify the effect of NAC on PD-1 inhibitor-induced cardiotoxicity, mice subjected to PD-1 inhibitor were administered NAC (Beyotime, Shanghai, China) at 400 mg/kg/day in their drinking water.

### 2.2 Echocardiography

For echocardiography, 28 days after the first administration of the PD-1 inhibitor, mice were anesthetized with 1.5%–2% isoflurane. After depilation, they were placed on a heating platform for heat preservation. Two-dimensional M-mode recordings were obtained using a Vevo 3100 LT high-resolution imaging system (VisualSonics, Toronto, Canada). Left ventricular ejection fraction (EF) and fractional shortening (FS) were recorded to evaluate cardiac function. Data measurement and analysis were performed on three consecutive cardiac cycles.

### 2.3 Histologic evaluation

The heart was harvested and fixed with 4% paraformaldehyde at 4°C for 24 h. The tissue was then dehydrated in ethanol, embedded in paraffin, and sectioned into 5 μm slices. For hematoxylin-eosin (HE), Masson’s trichrome staining, and Sirius Red staining, the heart slides were deparaffinized and rehydrated by gradient elution using xylene and ethanol, and then stained with HE staining reagent, Masson’s trichrome staining reagent, and Sirius Red staining reagent (Servicebio, Wuhan, China) according to the manufacturer’s instructions.

### 2.4 SnRNA-seq

Cardiac tissues were harvested from mice and washed in pre-cooled PBSE (PBS buffer containing 2 mM EGTA). Nuclei isolation was performed using GEXSCOPE^®^ Nucleus Separation Solution (Singleron Biotechnologies, Nanjing, China). The isolated nuclei were resuspended in PBSE. The concentration of single nucleus suspension was adjusted to 3–4 × 10^5^ nuclei/mL in PBS. The single nucleus suspension was then loaded onto a microfluidic chip (GEXSCOPE^®^ Single NucleusRNA-seq Kit, Singleron Biotechnologies), and snRNA-seq libraries were constructed according to the manufacturer’s instructions (Singleron Biotechnologies). Sequencing was performed on an Illumina novaseq 6,000 instrument with 150 bp paired end reads. Subsequent analyses, including cell composition proportion analysis, gene expression analysis, Kyoto Encyclopedia of Genes and Genomes (KEGG) analysis, CellphoneDB cell communication analysis, and transcription factor analysis, were performed using the sequencing data.

### 2.5 Western blot

Tissue samples or cells were lysed using radioimmunoprecipitation (RIPA) buffer containing a protease inhibitor to extract total proteins. The lysates were clarified by centrifugation to obtain the protein samples. These samples were then subjected to SDS-PAGE, transferred to a PVDF membrane, blocked with 10% skim milk for 2 h, and probed overnight with primary antibodies (Supplementary Table S1) at 4°C. The next day, after incubation with HRP-labeled secondary antibodies (Supplementary Table S1), the bands were visualized using an ultra-sensitive multifunctional imaging system (Invitrogen, Carlsbad, United States) and analyzed for grayscale intensity using ImageJ software. GAPDH was used as an internal control.

### 2.6 Quantitative reverse transcription-PCR (qRT-PCR)

Total RNA was extracted from tissues and cells using TRIzol reagent (Invitrogen, Carlsbad, United States). The RNA was then reverse transcribed to cDNA using the PrimeScript RT reagent Kit with gDNA Eraser (Takara, Kusatsu, Japan). Next, the cDNA was quantitatively amplified using the TB Green PCR Kit (Takara, Kusatsu, Japan). The results were presented relative to GAPDH expression using the 2^−ΔΔCq^ method. The sequences of the primers are listed in Supplementary Table S2.

### 2.7 Cell culture and transfection

Human cardiac fibroblasts (HCFs) and human coronary endothelial cells (HCAECs) were purchased from ScienCell Research Laboratories (Carlsbad, United States). HCFs were cultured in Fibroblast Medium-2 (FM-2) supplemented with 5% fetal bovine serum, 1% FCGS, and 1% penicillin/streptomycin. HCAECs were cultured in Endothelial Cell Medium (ECM) supplemented with 5% fetal bovine serum, 1% ECGS, and 1% penicillin/streptomycin. Cell morphology was observed using an inverted phase contrast microscope, and images were acquired with a camera. For PD-1 inhibitor treatment, the PD-1 inhibitor was added to the medium at a concentration of 5 μg/mL and incubated for 24 h. For TCF12 transfection, a plasmid overexpressing TCF12 was designed and synthesized by GenePharma Co., Ltd (Suzhou, China). The plasmid was transfected into HCAECs using Lipo8000 (Beyotime, Shanghai, China).

### 2.8 Immunofluorescence staining

For the immunofluorescent staining of heart sections, the sections were deparaffinized and rehydrated by gradient elution using xylene and ethanol. Antigen retrieval was performed in sodium citrate solution for 20 min at 180°C and 15 min at 160°C. For the immunofluorescent staining of cells, the cells were cultured in 24-well plates with glass slides, fixed with 4% paraformaldehyde for 15 min, and permeabilized with 0.1% Triton-X for 30 min. Prepared heart sections or cells were blocked with goat serum for 1 h at room temperature and then incubated with the primary antibody (Supplementary Table S1) overnight at 4°C. The slides were washed and incubated with the fluorescent-labeled secondary antibody (Supplementary Table S1) at room temperature for 1 h. Nuclei were stained using 4,6-diamidino-2-phenylindole (DAPI) for 10 min at room temperature. The stained heart sections or cells were observed using a confocal laser scanning microscope (Leica, Wetzlar, Germany) and analyzed using ImageJ software.

### 2.9 ELISA

The supernatant from HCAECs treated with a PD-1 inhibitor and/or a TCF12-overexpressing plasmid was collected. TGF-β1 levels in the cell supernatant were measured using a TGF-β1 ELISA Kit (Elabscience, Wuhan, China) following the manufacturer’s instructions.

### 2.10 Cell co-culture experiments

Conditional medium co-culture was performed as depicted in Figure 3H. HCAECs were exposed to drugs for 24 h, after which the medium was replaced with fresh culture medium and incubated for another 24 h. The conditioned medium was then collected and mixed with HCFs medium at a 1:1 ratio for HCFs culturing.

Transwell co-culture was conducted as shown in Figure 4N. A Transwell coculture system with a 0.4 µm pore size (Corning, Glendale, United States) was utilized. HCAECs were cultured in the upper chamber, while HCFs were cultured in the lower chamber. Following drug administration to HCAECs, both HCAECs and HCFs were cocultured for 24 h. Subsequently, the HCFs were used for relevant experiments.

### 2.11 Wound healing assay

For the scratch wound healing assay, HCAECs were scratched with 10 μL pipette tips and washed twice with Dulbecco’s Phosphate Buffered Saline (DPBS). Subsequently, the cells were cultured in ECM with no serum. Images were captured with a microscope at 0, 6, and 12 h.

### 2.12 Measurement of intracellular ROS level, malondialdehyde (MDA) content, and superoxide dismutase (SOD) activity

Intracellular ROS was measured by dihydroethidium (DHE) staining. According to the instructions of the DHE reagent (Beyotime, Shanghai, China), HCAECs were incubated with 5 μM DHE for 30 min at 37°C. The cells were then washed three times with PBS, and ROS levels were reflected by DHE fluorescence intensity. The cells were observed and images were captured under a confocal laser scanning microscope (Leica, Wetzlar, Germany) and analyzed using ImageJ software. Intracellular MDA content and SOD activity were measured using their respective assay kits according to the manufacturer’s instructions (Beyotime, Shanghai, China).

### 2.13 Measurement of soluble collagen content

Collagen production was detected using a Sircol soluble collagen assay kit (Biocolor, Carrickfergus, United Kingdom). Initially, 1 mL of cell culture supernatant was mixed with 200 μL of Isolation & Concentration Reagent and incubated at 4°C overnight. After centrifugation, 1 mL of supernatant was discarded. Sircol Dye Reagent (1 mL) was then added to each sample to form the collagen-dye complex. Following this, the complex was washed with Acid-Salt Wash Reagent and subsequently dissolved using Alkali Reagent. Finally, 200 μL of the resulting sample was transferred into a 96-well plate, and the absorbance at 555 nm was measured using a BioTek microplate reader (Richmond, United States).

### 2.14 Chromatin immunoprecipitation (ChIP)

ChIP assays were performed using the Chip Assay kit (Thermo Fisher Scientific, Rockford, United States). Cells were crosslinked with 1% formaldehyde for 10 min at room temperature and quenched with glycine. DNA was immunoprecipitated from the sonicated cell lysates using the TCF12 antibody and subjected to qPCR to amplify the TCF12 binding sites. The primer sequences are shown in Supplementary Table S3.

### 2.15 Statistics

Data were presented as mean ± SD. Statistical comparisons between two groups were conducted using Student’s t-tests, while one-way analysis of variance (ANOVA) was employed for comparisons involving three groups or more. The p value less than 0.05 was considered statistically significant.

## 3 Results

### 3.1 PD-1 inhibitor induces imbalanced collagen distribution in the mouse heart

**FIGURE 1:**
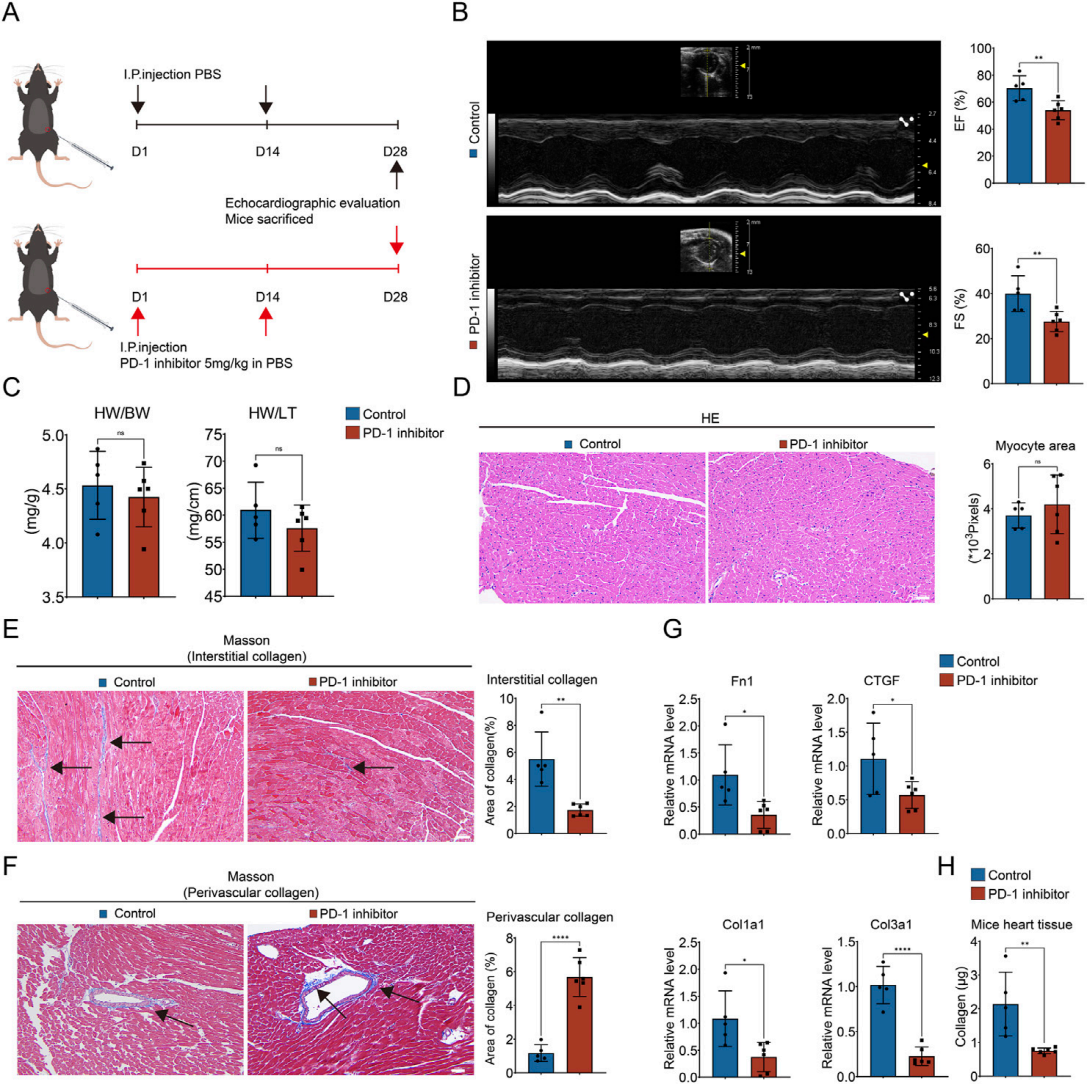
PD-1 inhibitor induces imbalanced collagen distribution in heart. **(A)** Schematic diagram of mouse model. 8-week-old male C57BL/6 mice were injected intraperitoneally with a PD-1 inhibitor on days 1 and 14 respectively, while the control mice were injected with an equal amount of PBS on the same day. Mice were sacrificed after performing echocardiography on day 28. **(B)** Representative echocardiography images and statistics of EF and FS of mouse heart. n = 5–6. **(C)** The ratio of heart weight to body weight (HW/BW) and heart weight to tibia length (HW/TL) of mice. n = 5–6. **(D)** Representative images of HE staining and quantification of myocyte area of mouse heart. n = 5–6. Scale bar: 100 μm. **(E)** Representative images of Masson’s trichrome staining and quantification of the interstitial collagen area of mouse heart. The black arrows indicate the region of collagen deposition. n = 5–6. Scale bar: 100 μm. **(F)** Representative images of Masson’s trichrome staining and quantification of the perivascular collagen area of mouse heart. The black arrows indicate the region of collagen deposition. n = 5–6. Scale bar: 100 μm. **(G)** Quantification of Fn1, CTGF, Col1a1, and Col3a1 mRNA levels in mouse heart using qRT-PCR. n = 5–6. **(H)** Measurement of the collagen content in mouse heart (values are shown per 10 mg of heart tissue). n = 5–6. *p < 0.05, **p < 0.01, ****p < 0.0001.

To study the toxic effect of the PD-1 inhibitor on the heart, we examined it from both functional and histological aspects. We established PD-1 inhibitor-induced cardiotoxicity mouse model based on a previous study (Xia et al., 2020). C57BL/6 mice were intraperitoneally injected with InVivoPlus anti-mouse PD-1 antibody at a dose of 5 mg/kg on the 1st and 14th days of a 28-day cycle (Figure 1A). Echocardiography showed that the left ventricular EF and FS in the PD-1 inhibitor group were significantly lower than those in the control group (Figure 1B). There was no significant difference in the ratios of heart weight to body weight and heart weight to tibia length (Figure 1C). Furthermore, we performed HE staining, Masson’s trichrome staining, and Sirius Red staining to observe the histological changes of the heart. No obvious structural changes of cardiomyocytes were observed in the heart treated with PD-1 inhibitor (Figure 1D). However, interestingly, we found that the PD-1 inhibitor led to an imbalanced collagen distribution, showing decreased interstitial collagen deposition and increased perivascular collagen deposition (Figures 1E, F; Supplementary Figures S1A, B). We also examined the expression of fibrosis-related genes and collagen content in the whole heart. The results showed that the PD-1 inhibitor induced decreased expression of Fn1, CTGF, Col1a1, and Col3a1 (Figure 1G) and decreased collagen content in the mouse heart compared with the control (Figure 1H). The above results indicate that collagen in the whole heart was reduced by PD-1 inhibitor treatment, but this reduction is not uniformly distributed throughout the heart, with interstitial collagen decreasing and perivascular collagen increasing.

### 3.2 PD-1 inhibitor does not directly influence CFs but affect the communication of other cardiac cells with CFs

**FIGURE 2:**
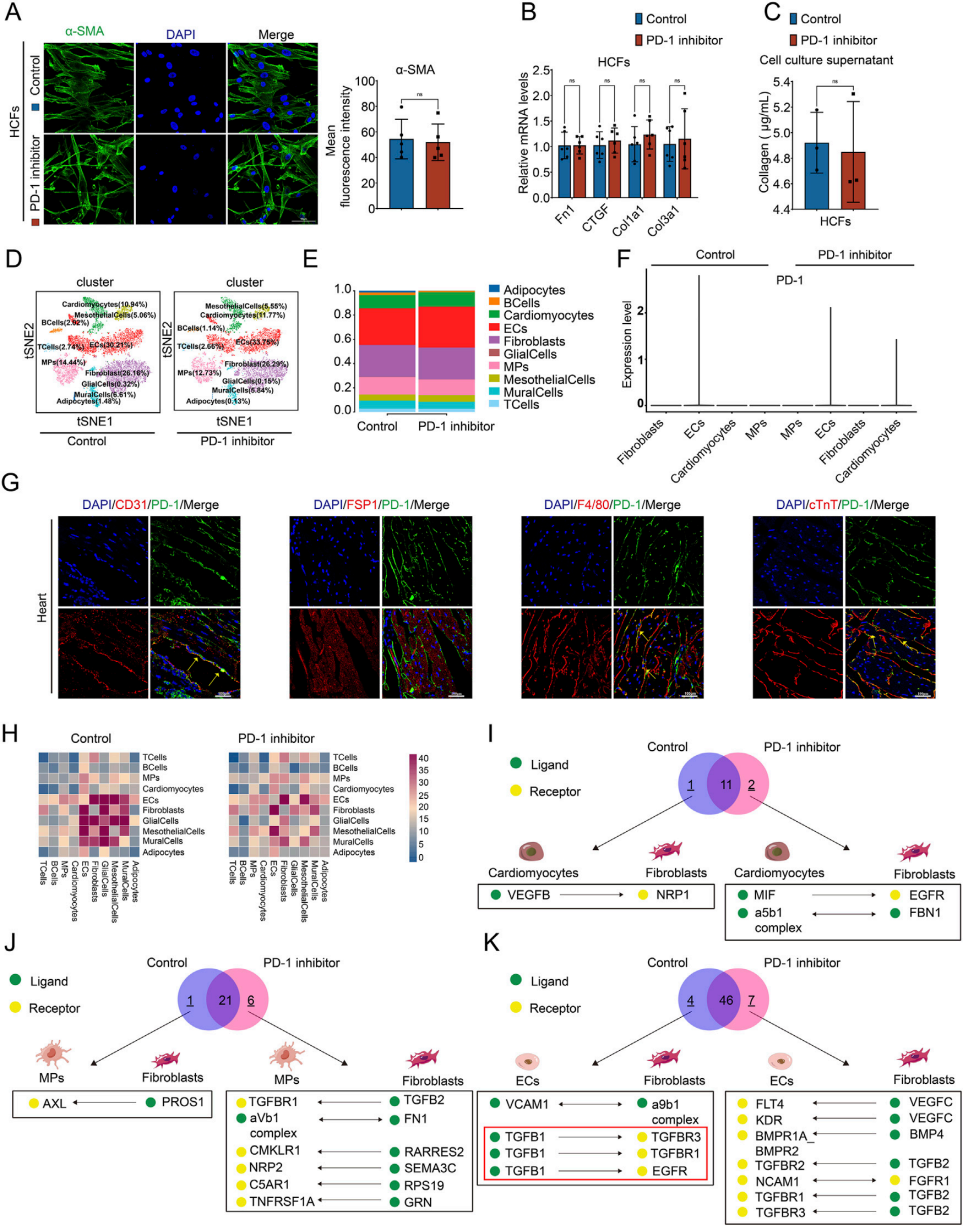
PD-1 inhibitor does not directly act on CFs but affect the communication of other cardiac cells with CFs. **(A)** Representative immunofluorescence images and quantification of the fluorescence intensity of α-SMA (green) in HCFs. Blue stands for DAPI. n = 5. Scale bar: 50 μm. **(B)** Quantification of Fn1, CTGF, Col1a1, and Col3a1 mRNA levels in HCFs using qRT-PCR. n = 6. **(C)** Measurement of the collagen content in cell culture supernatant of HCFs. n = 3. **(D)** t-distributed stochastic neighbor embedding (t-SNE) plot for snRNA-seq analysis in mouse hearts. Number indicates percentage of different cell clusters in heart. **(E)** The proportion of different cell clusters in mouse heart. **(F)** Violin plot showed the expression of PD-1 in Fibroblasts, ECs, Cardiomyocytes, and MPs in mouse hearts. **(G)** Representative immunofluorescence images of PD-1 (green) co-localized with different cell markers (red) in the mouse heart. CD31 represents ECs, FSP1 represents Fibroblasts, F4/80 represents MPs, and cTnT represents Cardiomyocytes. Scale bars: 100 μm. **(H)** CellphoneDB cell communication analysis showed the interaction counts between cell types. **(I-K)** Venn diagram showed the ligands-receptor interactions between Cardiomyocytes and Fibroblasts, MPs and Fibroblasts, and ECs and CFs in mouse heart. Black box showed interactions that present exclusively in the control group or the PD-1 group.

Firstly, to investigate the mechanism behind the imbalanced collagen distribution caused by PD-1 inhibitor, we examined the influence of PD-1 inhibitor on CFs, the primary cells responsible for the synthesis of extracellular matrix proteins (Burke et al., 2021; Frangogiannis, 2021; Liu et al., 2021). The upregulation of α-SMA is a sign of CFs activation and a marker of the fibroblast-to-myofibroblast transition (FMT) (Qin et al., 2015). HCFs were cultured and treated with the PD-1 inhibitor for 24 h. Immunofluorescence staining of α-SMA showed that compared to the control group, there was no significant difference in α-SMA expression in the PD-1 inhibitor-treated HCFs (Figure 2A). Additionally, the expression levels of fibrosis-related genes Fn1, CTGF, Col1a1, and Col3a1 in HCFs were not affected by PD-1 inhibitor treatment (Figure 2B). The cell culture supernatant from both control and PD-1 inhibitor-treated HCFs were collected. The collagen content measurement assay revealed no significant changes in collagen secretion between the control and PD-1 inhibitor-treated HCFs (Figure 2C). The above results indicated that PD-1 inhibitor does not directly influence the function of CFs. Therefore, we next performed snRNA-seq on the hearts of mice from both the control and PD-1 inhibitor groups to evaluate the effects of the PD-1 inhibitor on other cardiac cell types and the changes in their transcriptional profiles. 10 cell populations were identified in the heart, including Adipocytes, BCells, CMs, ECs, Fibroblasts, GlialCells, MPs, MesothelialCells, Mural Cells, and T Cells. Among these, ECs, Fibroblasts, MPs, and CMs were the most abundant (Figure 2D). The proportion of each cell type in the heart showed no significant changes by PD-1 inhibitor treatment (Figure 2E). Since PD-1 inhibitor may function through binding with PD-1, we examined the expression of PD-1 in the four major cell types in the heart. SnRNA-seq results showed PD-1 mainly expressed in ECs and CMs (Figure 2F) and immunofluorescence staining further confirmed that PD-1 was expressed in ECs, CMs, and MPs, but not in CFs (Figure 2G). This finding supports our results that PD-1 inhibitor does not directly affect CFs. Therefore, we proposed that PD-1 inhibitor might influence CFs through its effects on other cardiac cells via cell-cell crosstalk. To verify this hypothesis, we conducted cell-cell communication analysis using snRNA-seq data (Figure 2H). The ligand-receptor interactions between CM-CF, MP-CF, and EC-CF were analyzed, highlighting interactions present exclusively in the control group or the PD-1 inhibitor group, as shown in Figures 2I–K. Notably, three collagen synthesis-related interactions between ECs and CFs—TGFβ1_TGFβR1, TGFβ1_TGFβR3, and TGFβ1_EGFR—were absent in the hearts treated with PD-1 inhibitor. This observation led us to hypothesize that the reduced interstitial collagen deposition may result from changes in EC-CF crosstalk.

### 3.3 PD-1 inhibitor attenuates collagen synthesis in CFs by blocking the secretion of TGF-β1 from ECs

**FIGURE 3:**
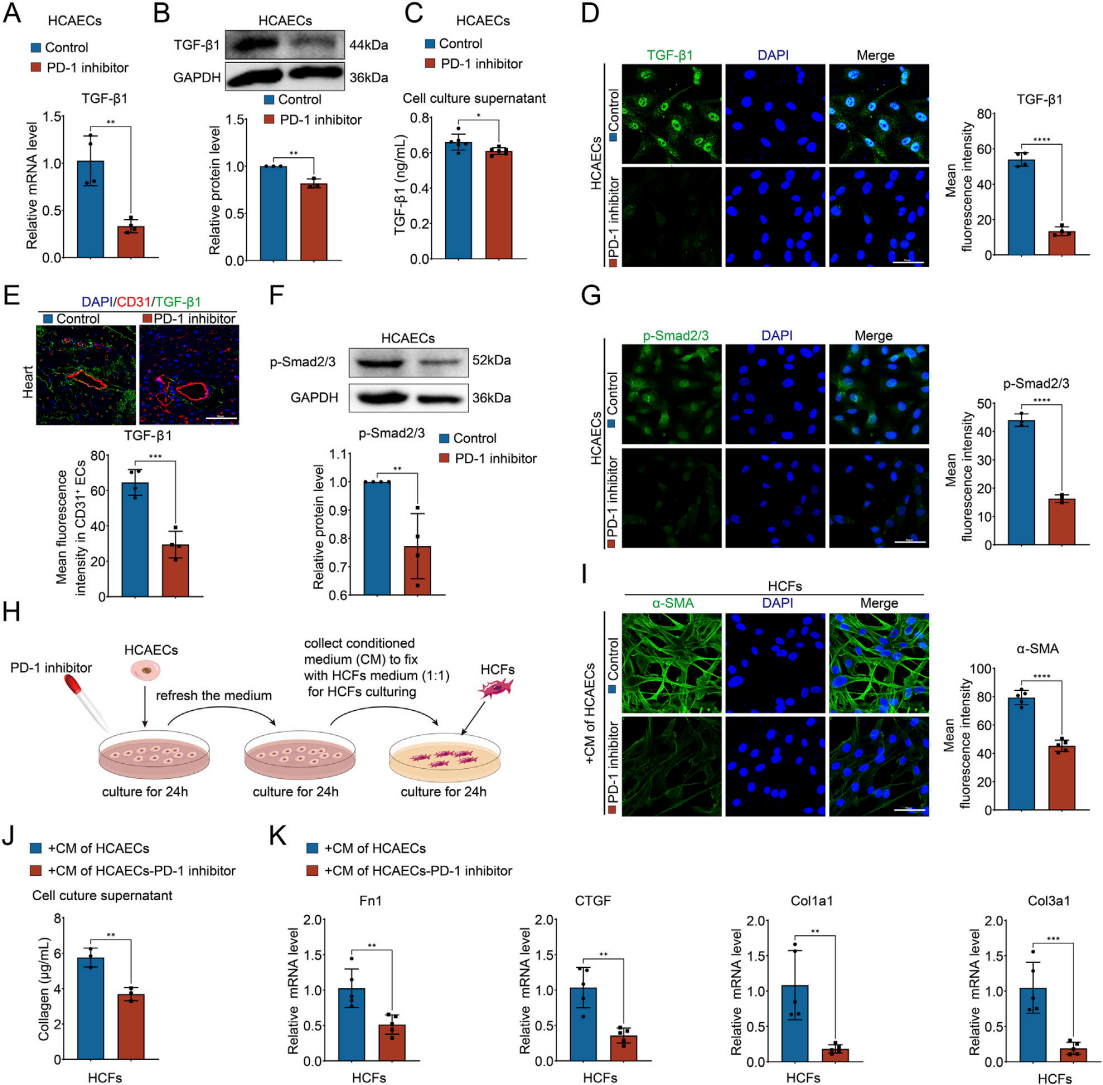
PD-1 inhibitor attenuates collagen synthesis in CFs by blocking the secretion of TGF-β1 from ECs. **(A)** Quantification of TGF-β1 mRNA level in HCAECs using qRT-PCR. n = 4. **(B)** Quantification of TGF-β1 protein level in HCAECs using Western blot. n = 3. **(C)** Measurement of the TGF-β1 level in cell culture supernatant of HCAECs using ELISA. n = 6. **(D)** Representative immunofluorescence images and quantification of the fluorescence intensity of TGF-β1 (green) in HCAECs. Blue stands for DAPI. n = 4. Scale bar: 50 μm. **(E)** Representative immunofluorescence images of CD31 (red) and TGF-β1 (green) in mouse heart and quantification of the fluorescence intensity of TGF-β1 in CD31^+^ ECs. Blue stands for DAPI. n = 4. Scale bar: 50 μm. **(F)** Quantification of p-Smad2/3 protein level in HCAECs using Western blot. n = 4. **(G)** Representative immunofluorescence images and quantification of the fluorescence intensity of p-Smad2/3 (green) in HCAECs. Blue stands for DAPI. n = 3. Scale bar: 50 μm. **(H)** Schematic diagram of the conditional medium co-culture system of HCAECs and HCFs. **(I)** Representative immunofluorescence images and quantification of the fluorescence intensity of α-SMA (green) in HCFs that cultured by the conditional medium of HCAECs. Blue stands for DAPI. n = 5. Scale bar: 50 μm. **(J)** Measurement of the collagen content in cell culture supernatant of HCFs that cultured by the conditional medium of HCAECs. n = 3. **(K)** Quantification of Fn1, CTGF, Col1a1, and Col3a1 mRNA levels in HCFs that cultured by the conditional medium of HCAECs using qRT-PCR. n = 5. *p < 0.05, **p < 0.01, ***p < 0.001, ****p < 0.0001.

Given that PD-1 inhibitor attenuated the interactions between ECs and CFs via TGF-β1 and its receptors, we investigated whether the secretion of TGF-β1 was reduced in PD-1 inhibitor-treated ECs. qRT-PCR and Western blot analysis revealed that both mRNA and protein levels of TGF-β1 in HCAECs were decreased by PD-1 inhibitor treatment (Figures 3A, B). Consistently, the secreted TGF-β1 in the cell culture supernatant was also reduced in PD-1 inhibitor-treated HCAECs (Figure 3C). Furthermore, both *in vitro* and *in vivo* immunofluorescence staining experiments confirmed that the expression of TGF-β1 in HCAECs and in CD31^+^ mouse cardiac ECs were decreased after PD-1 inhibitor administration (Figures 3D, E). Meanwhile, we also observed that the expression of phosphorylated Smad2/3 (p-Smad2/3), a downstream effector of TGF-β1 signaling, decreased in HCAECs following PD-1 inhibitor treatment (Figures 3F, G). These findings lead us to hypothesize that the reduction in TGF-β1 secretion by ECs, which subsequently affects CFs, may be the primary mechanism underlying the PD-1 inhibitor-induced decrease in cardiac interstitial collagen. To test this hypothesis, we conducted cell co-culture experiments using HCAECs and HCFs. The conditioned medium from PD-1 inhibitor-treated HCAECs was applied to HCFs, and the results indicated that this conditioned medium reduced the activity of HCFs, evidenced by reduced α-SMA expression (Figure 3I), decreased collagen secretion (Figure 3J), and lower expression levels of collagen synthesis-related genes Fn1, CTGF, Col1a1, and Col3a1 in HCFs (Figure 3K). These results confirm that the reduction in TGF-β1 secretion by ECs, which subsequently acts on CFs, is the primary mechanism underlying the PD-1 inhibitor-induced decrease in cardiac interstitial collagen.

### 3.4 PD-1 inhibitor decreases TGF-β1 expression by reducing TCF12 levels in ECs

**FIGURE 4:**
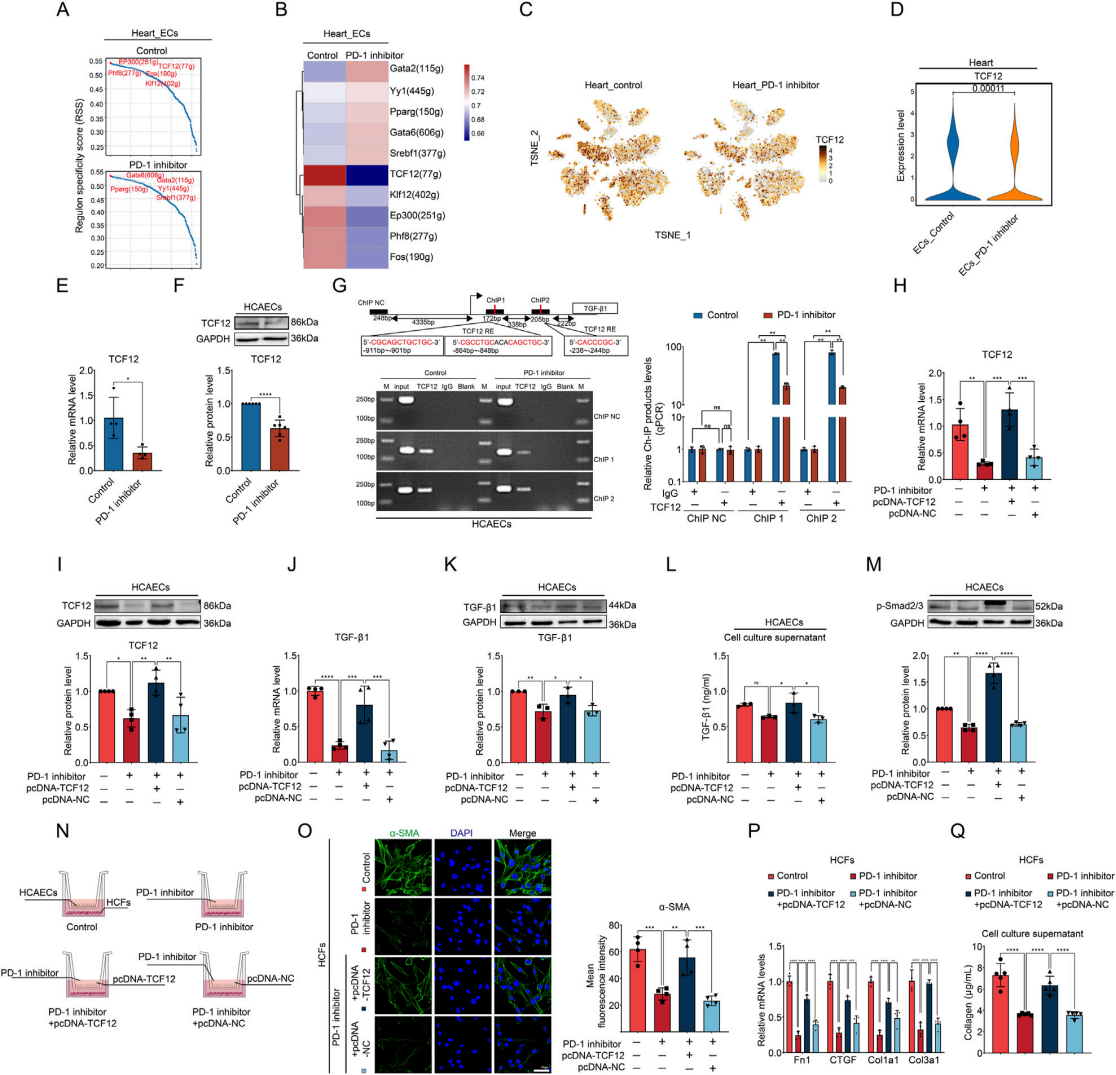
PD-1 inhibitor decreases TGF-β1 expression by reducing TCF12 in ECs. **(A, B)** Top 5 regulons with the highest regulon specificity scores (RSS) in cardiac ECs from control and PD-1 inhibitor-treated mouse hearts. **(C)** t-distributed stochastic neighbor embedding (t-SNE) plot showed the expression of TCF12 in different cell clusters in mouse heart. **(D)** Violin plot showed TCF12 levels in cardiac ECs from control and PD-1 inhibitor-treated hearts. **(E)** Quantification of TCF12 mRNA level in HCAECs using qRT-PCR. n = 4. **(F)** Quantification of TCF12 protein level in HCAECs using Western blot. n = 6. **(G)** Schematic diagram of the two putative binding elements of TCF12 within the TGF-β1 promoter region. TCF12-binding sites within the TGF-β1 promoter were detected in HCAECs by ChIP assay. n = 3. **(H)** Quantification of TCF12 mRNA level in HCAECs using qRT-PCR. n = 4. **(I)** Quantification of TCF12 protein level in HCAECs using Western blot. n = 4. **(J)** Quantification of TGF-β1 mRNA level in HCAECs using qRT-PCR. n = 4. **(K)** Quantification of TGF-β1 protein level in HCAECs using Western blot. n = 3. **(L)** Measurement of the TGF-β1 level in cell culture supernatant of HCAECs using ELISA. n = 3. **(M)** Quantification of p-Smad2/3 protein level in HCAECs using Western blot. n = 4. **(N)** Schematic diagram of the transwell co-culture system of HCAECs and HCFs. **(O)** Representative immunofluorescence images and quantification of the fluorescence intensity of α-SMA (green) in HCFs that co-cultured with HCAECs. Blue stands for DAPI. n = 4. Scale bar: 50 μm. **(P)** Quantification of Fn1, CTGF, Col1a1, and Col3a1 mRNA levels in HCFs that co-cultured with HCAECs using qRT-PCR. n = 4. **(Q)** Measurement of the collagen content in cell culture supernatant of HCFs that co-cultured with HCAECs. n = 5. *p < 0.05, **p < 0.01, ***p < 0.001, ****p < 0.0001.

To investigate the molecular mechanism by which PD-1 inhibitor reduces TGF-β1 expression in ECs, we analyzed the changes of transcription factor in ECs using snRNA-seq data. The top 5 regulons with the highest regulon specificity scores (RSS) in the control group and the PD-1 inhibitor group, respectively, are shown in Figures 4A, B. Notably, there was a significant reduction in RSS of TCF12 regulon in PD-1 inhibitor–treated ECs (Figure 4B), indicating the activity of TCF12 was reduced by PD-1 inhibitor. TCF12 is a transcription factor involved in the regulation of cell growth and differentiation (Yang et al., 2019). To determine if the reduced regulon activity of TCF12 is due to lower TCF12 expression, we investigated the impact of a PD-1 inhibitor on TCF12 levels. By analyzing snRNA-seq data, we observed a decrease in TCF12 expression following PD-1 inhibitor treatment across various heart cell types, including ECs (Figures 4C, D). This finding was further validated by qRT-PCR and Western blot analyses (Figures 4E, F). Studies have demonstrated that TCF12 can regulate TGF-β2 and contribute to melanoma tumorigenesis (Tian et al., 2023). However, there are no reports investigating the relationship between TCF12 and TGF-β1. Therefore, we experimentally investigated whether TCF12 binds to the promoter regions of TGF-β1 using a ChIP assay. TCF12 immunoprecipitates were found to be highly enriched in the TGF-β1 promoter region, and PD-1 inhibitor treatment resulted in a significant decrease in this binding (Figure 4G). To assess the regulatory function of TCF12 on TGF-β1, we overexpressed TCF12 using plasmid in PD-1 inhibitor-treated HCAECs and confirmed successful overexpression (Figures 4H, I). Results from qRT-PCR and Western blot analyses indicated that TCF12 overexpression effectively counteracted the inhibitory effect of the PD-1 inhibitor on TGF-β1 expression (Figures 4J, K) and secretion (Figure 4L) in HCAECs. Furthermore, the decreased level of p-Smad2/3 induced by the PD-1 inhibitor was also reversed following TCF12 overexpression (Figure 4M).

To investigate whether TCF12 overexpression in HCAECs could counteract the effects of the PD-1 inhibitor on EC-CF crosstalk, we performed cell co-culture experiments using the Transwell system (Figure 4N). Compared to HCFs co-cultured with PD-1 inhibitor-treated HCAECs, HCFs co-cultured with HCAECs treated with both the PD-1 inhibitor and TCF12 overexpression plasmid showed significantly increased activity, evidenced by increased α-SMA expression (Figure 4O), collagen synthesis-related gene expression (Figure 4P), and collagen secretion (Figure 4Q). These results suggest that PD-1 inhibitor affects EC-CF crosstalk and fibroblast activity by modulating TCF12/TGF-β1 axis in ECs.

### 3.5 PD-1 inhibitor induces EndMT in ECs

**FIGURE 5:**
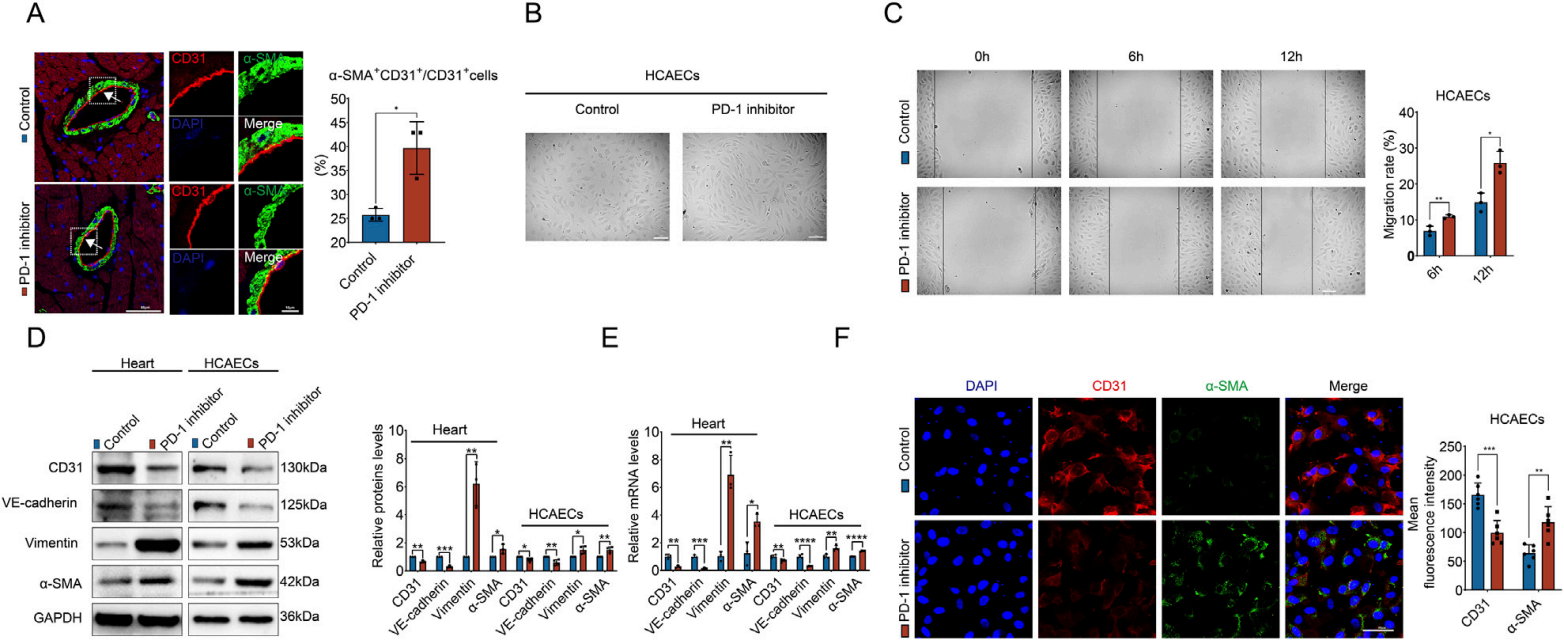
PD-1 inhibitor induces EndMT in ECs. **(A)** Representative immunofluorescence images of CD31 (red) and α-SMA (green) in mouse heart, and quantification of the percentage of α-SMA^+^CD31^+^ cells relative to CD31^+^ cells. Blue stands for DAPI. n = 3. Scale bars: 50 μm and 10 μm. **(B)** Representative images of cell morphology of HCAECs treated with PD-1 inhibitor or not. Scale bars: 100 μm. **(C)** Measurement of the migration of HCAECs by wound healing assay. n = 3. Scale bar: 100 μm. **(D)** Quantification of protein levels of EndMT-related markers (CD31, VE-cadherin, Vimentin, and α-SMA) in mouse hearts and HCAECs using Western blot. n = 3–4. **(E)** Quantification of mRNA levels of EndMT-related markers (CD31, VE-cadherin, Vimentin, and α-SMA) in mouse hearts and HCAECs using qRT-PCR. n = 3–4. **(F)** Representative immunofluorescence images and quantification of the fluorescence intensity of CD31 (red) and α-SMA (green) in HCAECs. Blue stands for DAPI. n = 6. Scale bar: 50 μm *p < 0.05, **p < 0.01, ***p < 0.001, ****p < 0.0001.

EndMT has been found to cause ECs to secrete collagen by transforming into EndMT-derived fibroblasts in the heart. Therefore, we generated an idea that PD-1 inhibitor may promote perivascular fibrosis through inducing EndMT. To investigate this, we performed immunofluorescence staining to examine EndMT markers (endothelial marker CD31 and mesenchymal marker α-SMA) in heart. The results showed that CD31^+^α-SMA^+^ cells were prominent in the coronary artery intima in the PD-1 inhibitor group, whereas such double-positive cells were rarely detectable in the control group (Figure 5A), indicating EndMT occurred in PD-1 inhibitor-treated heart. Furthermore, following PD-1 inhibitor treatment, HCAECs transitioned from a cobblestone-like endothelial phenotype to a spindle-shaped mesenchymal phenotype (Figure 5B). Wound healing assay demonstrated enhanced migratory ability of HCAECs after PD-1 inhibitor treatment (Figure 5C). Additionally, as shown in Figures 5D, E, Western blot and qRT-PCR analyses revealed that PD-1 inhibitor significantly reduced the expression of endothelial markers (CD31 and VE-cadherin) while upregulating the expression of mesenchymal markers (α-SMA and Vimentin) in both mouse heart and HCAECs. Immunofluorescence staining further confirmed that PD-1 inhibitor-treated HCAECs underwent EndMT (Figure 5F). Collectively, these results indicate that PD-1 inhibitor induces EndMT in ECs, which may be responsible for the increased perivascular collagen deposition observed in PD-1 inhibitor-treated hearts.

### 3.6 PD-1 inhibitor-induced endothelial dysfunction is associated with ROS accumulation

**FIGURE 6:**
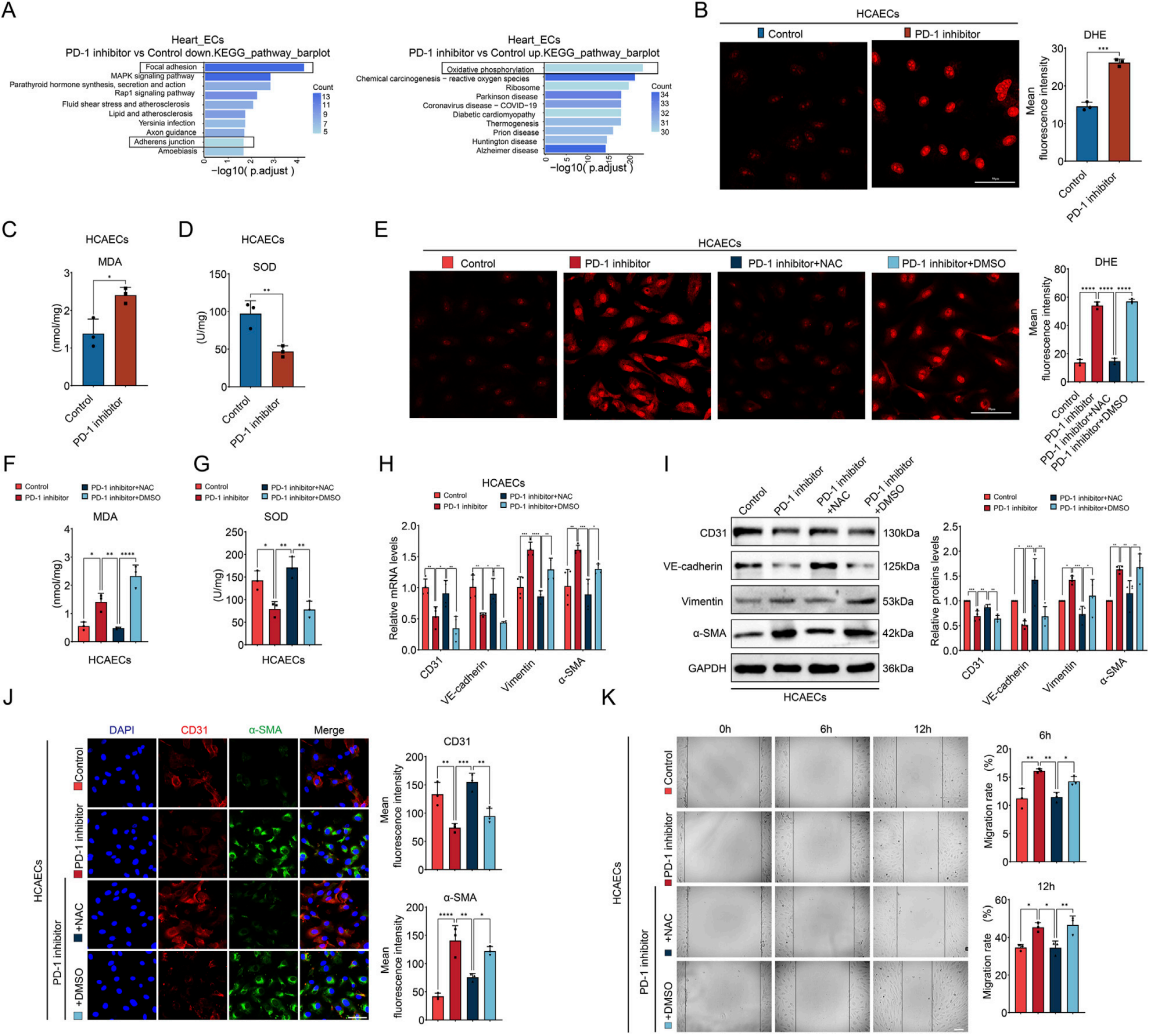
PD-1 inhibitor-induced endothelial dysfunction is associated with ROS accumulation. **(A)** The KEGG analysis of DEGs in ECs of mouse heart after treatment with PD-1 inhibitor. **(B)** Representative fluorescence images and quantification of the fluorescence intensity of DHE (red) in HCAECs. n = 3. Scale bar: 50 μm. **(C)** Measurement of the MDA content in HCAECs. n = 3. **(D)** Measurement of the SOD activity in HCAECs. n = 3. **(E)** Representative fluorescence images and quantification of the fluorescence intensity of DHE (red) in HCAECs. n = 3. Scale bar: 50 μm. **(F)** Measurement of the MDA content in HCAECs. n = 3. **(G)** Measurement of the SOD activity in HCAECs. n = 3. **(H)** Quantification of mRNA levels of EndMT-related markers (CD31, VE-cadherin, Vimentin, and α-SMA) in HCAECs using qRT-PCR. n = 4. **(I)** Quantification of protein levels of EndMT-related markers (CD31, VE-cadherin, Vimentin, and α-SMA) in HCAECs using Western blot. n = 4. **(J)** Representative immunofluorescence images and quantification of the fluorescence intensity of CD31 (red) and α-SMA (green) in HCAECs. Blue stands for DAPI. n = 3. Scale bar: 50 μm. **(K)** Measurement of the migration of HCAECs by wound healing assay. n = 3. Scale bar: 100 μm *p < 0.05, **p < 0.01, ***p < 0.001, ****p < 0.0001.

From the above findings, it can be seen that PD-1 inhibitor primarily acts on cardiac ECs, affecting EC-CF communication and EndMT. Therefore, we want to further explore the reasons behind the effects of PD-1 inhibitor on ECs. We made KEGG pathway analysis of differently expressed genes (DEGs) in cardiac ECs based on the snRNA-seq data. KEGG pathway analysis on the downregulated genes revealed significant enrichment in pathways related to focal adhesion and adherens junction (Figure 6A). These pathways play critical roles in maintaining cellular structure and function, and they are intricately involved in the process of EndMT (Ciszewski et al., 2021). EndMT are characterized by a phenotypic switch involving the breakdown of both focal adhesions and adherens junctions, allowing ECs to detach from their neighbors and the extracellular matrix, thereby migrating and invading surrounding tissues (Alvandi and Bischoff, 2021; Wiktorska et al., 2023). Therefore, this result supports our finding that PD-1 inhibitor induces EndMT in ECs. KEGG pathway analysis on the upregulated genes revealed that the oxidative phosphorylation pathway was significantly enriched (Figure 6A). The overactivation of oxidative phosphorylation can lead to an increase in ROS, which acts as a damaging factor to ECs (Sobierajska et al., 2022; Thuan et al., 2018). Therefore, we hypothesized that ROS accumulation might be responsible for the PD-1 inhibitor-induced endothelial dysfunction. To test this hypothesis, we first used DHE staining to detect ROS levels within cells. The results showed that the PD-1 inhibitor significantly increased ROS and MDA levels (Figures 6B, C) and decreased the activity of the antioxidant enzyme SOD in HCAECs (Figure 6D), indicating that the PD-1 inhibitor caused ROS accumulation and oxidative stress in ECs. Next, we used the ROS inhibitor NAC to assess whether ROS scavenging could reverse PD-1 inhibitor-induced EndMT. As shown in Figures 6E–G, the addition of NAC successfully inhibited ROS accumulation and oxidative stress induced by the PD-1 inhibitor. Importantly, the addition of NAC reversed PD-1 inhibitor-induced EndMT, as evidenced by increased expression of endothelial markers and decreased expression of mesenchymal markers in the NAC treatment group (Figures 6H–J). The wound healing assay confirmed that NAC treatment reversed the increased cell migratory ability induced by PD-1 inhibitor (Figure 6K). Overall, these results indicate that PD-1 inhibitor induces endothelial dysfunction via causing ROS accumulation.

### 3.7 NAC supplementation reverses PD-1 inhibitor-induced cardiac dysfunction and restores balanced collagen distribution

**FIGURE 7:**
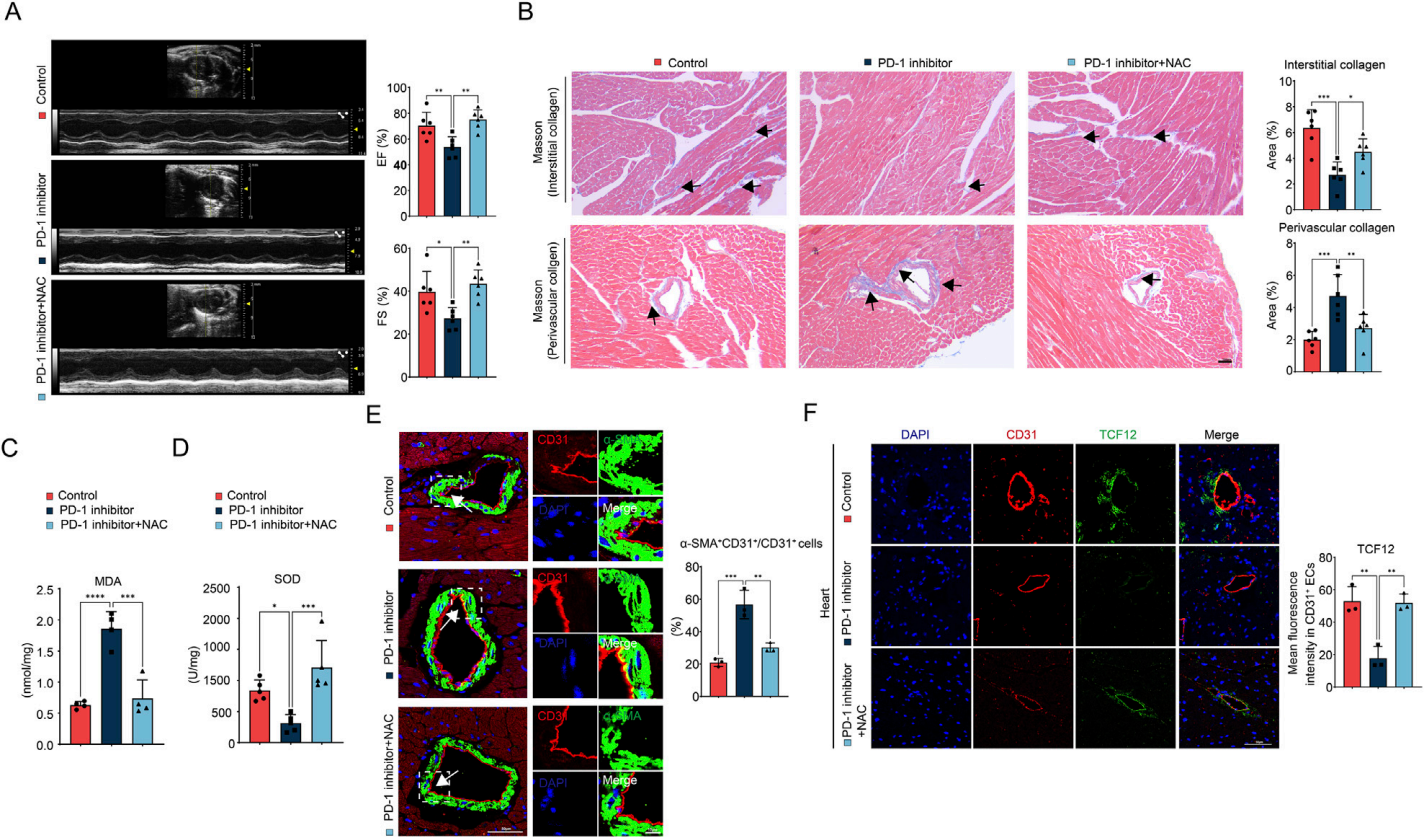
NAC supplementation ameliorates PD-1 inhibitor-induced cardiac dysfunction and imbalanced collagen distribution. **(A)** Representative echocardiography images and statistics of EF and FS of mouse heart. n = 6. **(B)** Representative images of Masson’s trichrome staining and quantification of the interstitial and perivascular collagen area of mouse heart. The black arrows indicate the region of collagen deposition. n = 6. Scale bar: 100 μm. **(C)** Measurement of the MDA content in mouse heart. n = 4. **(D)** Measurement of the SOD activity in mouse heart. n = 5. **(E)** Representative immunofluorescence images of CD31 (red) and α-SMA (green) in mouse heart and quantification of the percentage of α-SMA^+^CD31^+^ cells relative to CD31^+^ cells. Blue stands for DAPI. n = 3. Scale bar: 50 μm. **(F)** Representative immunofluorescence images of CD31 (red) and TCF12 (green) in mouse heart and quantification of the fluorescence intensity of TCF12 in CD31^+^ ECs. Blue stands for DAPI. n = 3. Scale bar: 50 μm *p < 0.05, **p < 0.01, ***p < 0.001, ****p < 0.0001.

To further validate the effects of NAC supplementation on PD-1 inhibitor-induced cardiac dysfunction and imbalanced collagen distribution *in vivo*, we administered oral NAC (400 mg/kg/day in their drinking water) to mice. The results showed that NAC supplementation reversed the cardiac dysfunction caused by the PD-1 inhibitor (Figure 7A). Additionally, NAC supplementation also reversed the increased perivascular collagen distribution and reduced interstitial collagen distribution induced by the PD-1 inhibitor (Figure 7B; Supplementary Figures S2A, B). NAC supplementation mitigated the PD-1 inhibitor-induced ROS accumulation and oxidative stress (Figures 7C, D), as well as EndMT, as evidenced by the decreased number of CD31^+^α-SMA^+^ cells in the coronary artery intima in the PD-1 inhibitor + NAC group (Figure 7E). Immunofluorescence staining also revealed that the decreased expression of TCF12 in coronary artery intima induced by PD-1 inhibitor was also reversed by NAC supplementation (Figure 7F). In summary, we conclude that NAC supplementation can reverse PD-1 inhibitor-induced cardiac dysfunction and restore balanced cardiac collagen distribution. This effect is achieved by inhibiting EndMT and restoring TCF12 expression in cardiac ECs.

## 4 Discussion

Cancer immunotherapy, encompassing checkpoint inhibitors and adoptive cell therapy, harnesses the immune system to identify and target cancer cells (Kennedy and Salama, 2020). Compared to traditional radiotherapy and chemotherapy, immunotherapy exhibits reduced toxicity and fewer side effects. PD-1 inhibitors bind to the PD-1 receptor on T cells, reactivating them and enabling them to recognize and attack cancer cells. These inhibitors initially approved for the treatment of stage IV malignant melanoma, but are now successfully used in various cancers, such as non-small-cell lung cancer and lymphoma (Shiravand et al., 2022). However, along with the increased use, the incidence of cardiac immune-related adverse events (irAEs) has risen and become a social concern (Wu et al., 2024). A large variety of cardiotoxic events with manifestations such as myocarditis, heart failure, cardiomyopathy, heart block, and myocardial fibrosis were documented in PD-1 blocking immunotherapy (Liu et al., 2022). Once cardiotoxicity occurs, its fatality rate is extremely high (39.7%–50% for myocarditis) (Salem et al., 2018). Therefore, investigating early cardiac pathological changes and preventing severe complications induced by PD-1 inhibitors is extremely important. However, the understanding of the mechanism behind PD-1 inhibitor-induced cardiotoxicity is limited. Our research results revealed that cardiac function in mice significantly decreased 4 weeks after the first treatment of PD-1 inhibitor (two cycles), with reductions in both EF and FS, which is consistent with previous studies (Xia et al., 2020).

Myocardial fibrosis is characterized by an increase in extracellular matrix proteins, myocardial cell disarray, and heart structure remodeling (Russo and Frangogiannis, 2016). Myocardial fibrosis is frequently reported in clinical cases of ICI-induced cardiotoxicity. This fibrosis can occur alongside myocarditis, myocardial hypertrophy, or as an isolated finding without other associated myocardial changes (Heinzerling et al., 2016). Some cases of fibrosis occurring without myocarditis suggest that ICI may directly affect collagen distribution in the heart. However, compared to myocarditis, our understanding of the mechanisms underlying ICI-induced myocardial fibrosis is still very limited.

Surprisingly, our histological results revealed that PD-1 inhibitor did not induce simple myocardial fibrosis but led to an overall decrease in collagen content in the heart. However, collagen was not uniformly reduced; instead, there was decreased interstitial collagen deposition and increased perivascular collagen deposition, indicative of typical perivascular fibrosis and a deficiency in interstitial collagen. This phenomenon has not been reported yet. The cardiac interstitial collagen forms an extracellular matrix network in the heart that provides mechanical support to CMs, helping to maintain the stability and elasticity of myocardial tissue. Therefore, a reduction in cardiac interstitial collagen weakens its support for CMs, leading to impaired myocardial conduction and functional dysfunction. Perivascular collagen in the heart is primarily located near the vascular basement membrane and pericytes, maintaining the shape and elasticity of blood vessels. An increase in perivascular collagen can lead to vascular stiffness, which further impacts cardiac blood supply, ultimately resulting in decreased cardiac function. Therefore, this remodeling may be the pathological basis for the deterioration of cardiac function induced by PD-1 inhibitor, potentially leading to reduced cardiac compliance, impaired cardiac function, arrhythmia, heart failure, and sudden cardiac death.

Interestingly, we found that PD-1 inhibitor does not directly affect CFs, the main cells responsible for collagen secretion in the heart. Therefore, we focused on exploring the target cells of PD-1 inhibitor in the heart and their potential effects on CFs. In recent years, single-cell sequencing has been widely used and plays an important role in the study of cardiac collagen remodeling. For example, studies have revealed the key role of CTHRC1 in collagen deposition after myocardial infarction by single-cell sequencing (Ruiz-Villalba et al., 2020). There has also been progress in using single-cell sequencing to analyze key changes in ligand-receptor interactions between different fibroblast subtypes that drive cardiac collagen production in a diabetic mouse model (Li et al., 2023). In this study, we use snRNA-seq to explore the effects of PD-1 inhibitor on different cardiac cells. Through single gene mapping, we found that the expression of PD-1 in cardiac ECs was higher than that in other types of cells, indicating that PD-1 inhibitors may act on ECs and cause cardiotoxicity. ECs are the largest endocrine organs in the body and can influence the function of other cardiac cells, including CFs, through the secretion of cytokines (Kongpol et al., 2019; Li Y. et al., 2024). Cell communication analysis revealed that ligand-receptor interactions between ECs and CFs were altered by PD-1 inhibitor treatment. Specifically, three collagen synthesis-related ligand-receptor pairs—TGFβ1_EGFR, TGFβ1_TGFβR3, and TGFβ1_TGFβR1—were absent in the heart treated with the PD-1 inhibitor. Further experiments demonstrated that PD-1 inhibitor treatment downregulated TGF-β1 expression in cardiac ECs, resulting in reduced TGF-β1 secretion. When CFs were cultured in conditioned medium containing EC secretions, it was observed that ECs treated with the PD-1 inhibitor induced a decrease in collagen secretion by CFs. In contrast, directly treating CFs with the PD-1 inhibitor did not affect their collagen secretion. Therefore, the reduced secretion of TGF-β1 by ECs, which subsequently affects CFs, contributes to the decrease in cardiac interstitial collagen observed after PD-1 inhibitor administration.

Transcription factor TCF12, is a member of the basic Helix-Loop-Helix protein (bHLH) protein family, proven to play an important role in different developmental processes like neurogenesis, hematopoietic specification, and T cell development (Barndt et al., 2000; Mesman and Smidt, 2017). Recent studies have discovered that TCF12 is involved in tumorigenesis, such as inducing ferroptosis in oral squamous cell carcinoma (Liu et al., 2024) and promoting angiogenesis in osteosarcoma (Li W. et al., 2024); however, its role in cardiovascular diseases has been less explored. Our findings indicate that the PD-1 inhibitor-induced downregulation of TCF12 is responsible for the reduced secretion of TGF-β1 by ECs, with TGF-β1 being a direct transcriptional target of TCF12.

EndMT is a process where ECs lose their endothelial traits and acquire mesenchymal characteristics. This process involves the loss of endothelial markers like CD31 and VE-cadherin, a reduction in endothelial function like tube formation capability, and the gain of mesenchymal markers such as α-SMA and FSP1, along with mesenchymal functions like collagen secretion (Hall et al., 2024). Zeiberg et al. first identified EndMT in a myocardial fibrosis model induced by pressure overload, discovering that EndMT-derived myofibroblasts comprised 27%–35% of the fibrotic myocardium (Zeisberg et al., 2007). In this study, since perivascular fibroblasts may largely depend on EC-derived TGF-β and thus exhibit reduced collagen synthesis, the observed increase in perivascular collagen distribution induced by PD-1 inhibitor led us to consider the involvement of EndMT. Indeed, our results from both *in vivo* and *in vitro* experiments demonstrated that EndMT occurs upon PD-1 inhibitor administration. Therefore, the increase in perivascular collagen deposition is primarily attributed to enhanced collagen secretion from EndMT-transformed ECs.

Furthermore, we found that PD-1 inhibitor significantly activated oxidative phosphorylation pathway, and excessive activation of oxidative phosphorylation could induce the increase of ROS (Nolfi-Donegan et al., 2020). In this study, we found that the level of ROS in ECs was significantly increased by PD-1 inhibitor. NAC, a ROS inhibitor with antioxidant and anti-inflammatory activity, is a drug approved by food and drug administration (FDA) for acetaminophen overdose treatment and, more recently, as a mucolytic agent in respiratory diseases (Tenorio et al., 2021) and an adjunct medication for neurodegenerative diseases (Tardiolo et al., 2018). In some countries, NAC is widely available over-the-counter as a nutritional supplement. In this study, we proved that NAC supplementation in mice could reverse PD-1 inhibitor-induced cardiac dysfunction and restore balanced collagen distribution. NAC supplementation could inhibit PD-1 inhibitor-induced EndMT in cardiac ECs, and it could also reverse the decreased expression of TCF12 in ECs. Thus, by inhibiting EndMT and TCF12 downregulation, NAC alleviates both increased perivascular collagen deposition and decreased interstitial collagen, restores cardiac collagen distribution balance, and reverses the cardiac function decline caused by PD-1 inhibitor.

Notably, our study appears to reveal a contradiction between the decreased TGF-β production in ECs and the observed EndMT, as TGF-β is a major inducer of EndMT. Therefore, we suggest that ROS accumulation serves as the primary driver of EndMT in PD-1 inhibitor-treated ECs, independent of TGF-β upregulation. The specific pathways through which ROS induces EndMT in PD-1 inhibitor-treated ECs will be the focus of our future investigations. Additionally, since exosomes are small extracellular vesicles that play a pivotal role in cell-to-cell communication (Zhao et al., 2024), it is interesting to explore whether TGF-β1, as a mediator in EC-CF crosstalk, is transported through exosomes. It is also interesting to investigate whether other exosome-carried mRNA, non-coding RNAs (ncRNAs), lipids, or proteins are involved in PD-1 inhibitor-induced EC-CF crosstalk. Indeed, regarding PD-1 inhibitor-induced cardiotoxicity, Xia et al. has found that exosomes derived from PD-1 inhibitor-treated macrophages exerted a pro-senescent effect on cardiomyocytes, with miR-34a-5p identified as an exosomal transfer RNA (Xia et al., 2020). Therefore, we will investigate the involvement of exsomes in PD-1 inhibitor-induced cardiac collagen imbalance in our future research.

In summary, our study presents a previously unreported cardiotoxicity of PD-1 inhibitor: an imbalance in collagen distribution within the heart, characterized by decreased interstitial collagen deposition and increased perivascular collagen deposition. Mechanistically, PD-1 inhibitor targets cardiac ECs, leading to ROS accumulation, which causes weakened EC-CF crosstalk and EndMT occurrence. NAC supplementation effectively restores balanced collagen distribution and prevents cardiac dysfunction in PD-1 inhibitor-treated mice. These findings suggest that NAC supplementation could potentially be an effective clinical strategy to mitigate PD-1 inhibitor-induced cardiotoxicity.

## Data Availability

The datasets presented in this study can be found in an online repository. The name of the repository is https://www.ncbi.nlm.nih.gov/geo/ GSE284718.
