# Supplementary material for: PD-1 inhibition disrupts collagen homeostasis and aggravates cardiac dysfunction through endothelial-fibroblast crosstalk and EndMT
Source: Front Pharmacol. 2025 Mar 17;16:1549487. doi: 10.3389/fphar.2025.1549487 (PMC11955664; doi:10.3389/fphar.2025.1549487)
Supplement: Supplementary file 1 [file Table1.DOCX]

Supplementary Material

PD-1 inhibition disrupts collagen homeostasis and aggravates cardiac dysfunction through endothelial-fibroblast crosstalk and EndMT

Zejin Zhang, Zhenzhen Yan, Tao Yuan, Xiaona Zhao, Minghui Wang, Guoqing Liu, Lijun Gan, Wei Qin

**
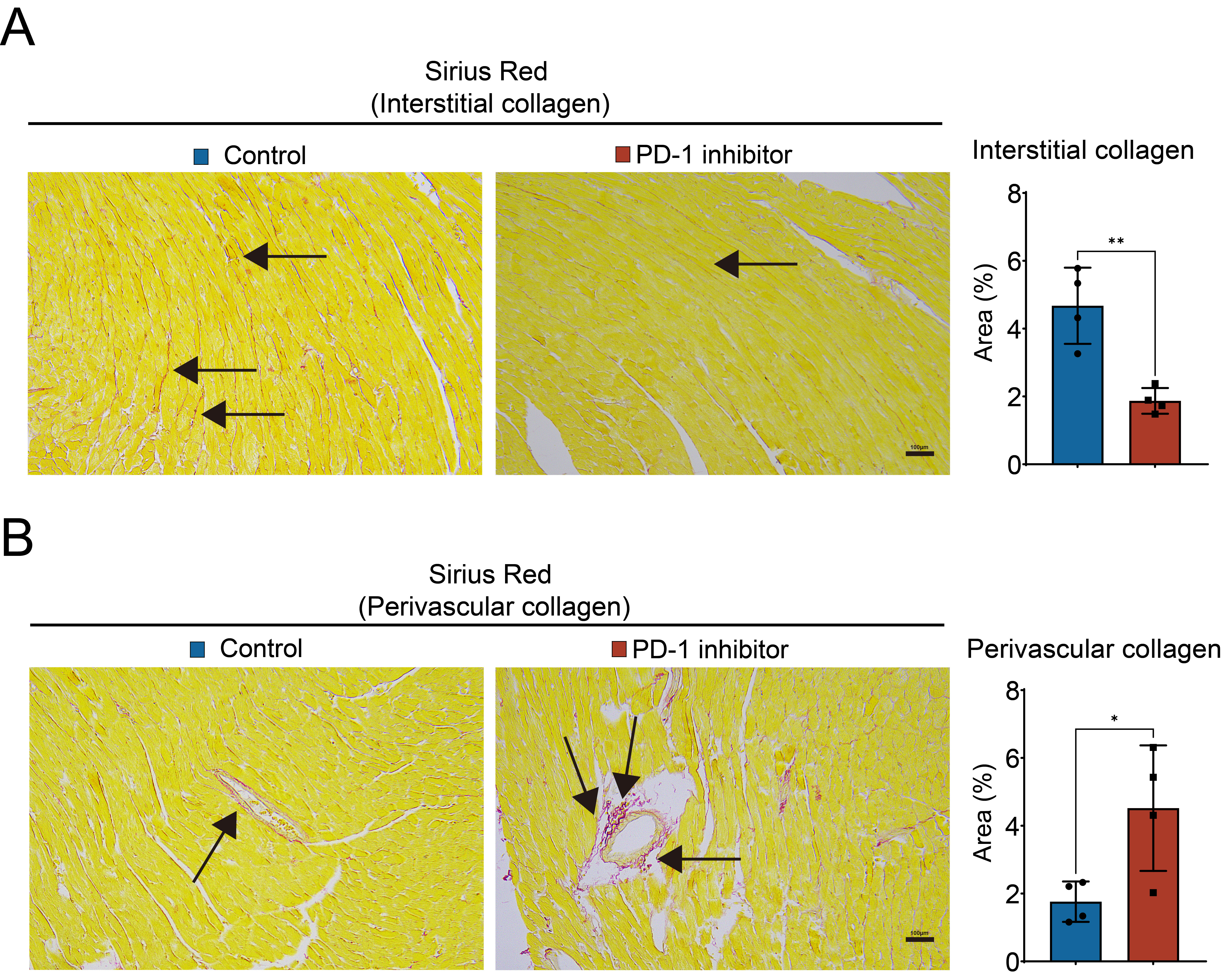
**

**Supplementary Figure 1 PD-1 inhibitor induces imbalanced collagen distribution in heart detected by Sirius Red staining. A** Representative images of Sirius Red staining and quantification of the interstitial collagen area of mouse heart. The black arrows indicate the region of collagen deposition. n=4. Scale bar: 100 μm. **B** Representative images of Sirius Red staining and quantification of the perivascular collagen area of mouse heart. The black arrows indicate the region of collagen deposition. n=4. Scale bar: 100 μm. **p*<0.05, ***p*<0.01.

**
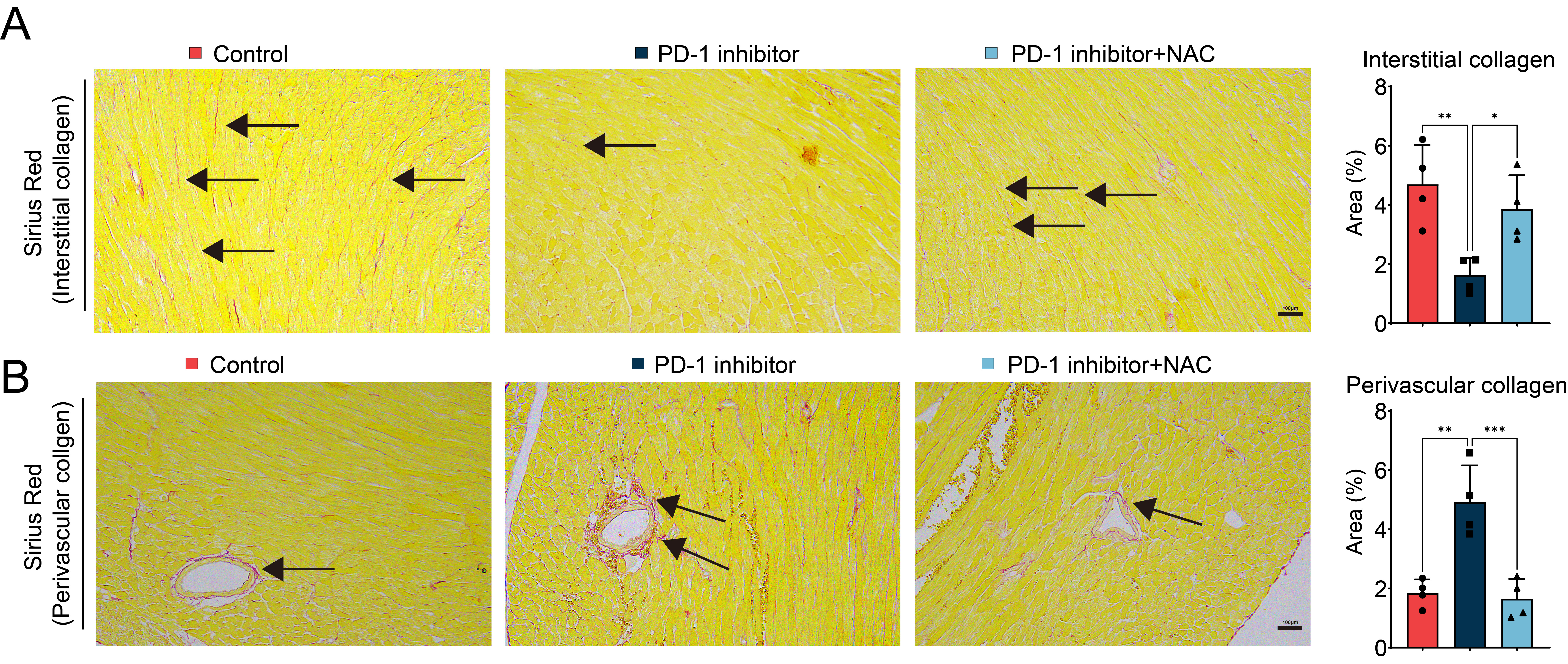
**

**Supplementary Figure 2 NAC supplementation ameliorates PD-1 inhibitor-induced collagen imbalance in heart detected by Sirius Red staining.** **A** Representative images of Sirius Red staining and quantification of the interstitial collagen area of mouse heart. The black arrows indicate the region of collagen deposition. n=4. Scale bar: 100 μm. **B** Representative images of Sirius Red staining and quantification of the perivascular collagen area of mouse heart. The black arrows indicate the region of collagen deposition. n=4. Scale bar: 100 μm. **p*<0.05, ***p*<0.01, ****p*<0.001.

**Table S1 Antibodies used in the experiments**

| **Antibody** | **Source** | **Company** | **Catalog No.** |
| --- | --- | --- | --- |
| Anti-PD-1/CD279 | Mouse | Proteintech | 66220-1-lg |
| Anti-F4/80 | Rabbit | Proteintech | 28463-1-AP |
| Anti-Cardiac Troponin T | Rabbit | Proteintech | 15513-1-AP |
| Anti-p-SMAD2/3 | Rabbit | Wanleibio | WL02305 |
| Anti-TCF12 | Rabbit | Cell Signaling Technology | 11825 |
| Anti-TGF-β1 | Rabbit | Proteintech | 21898-1-AP |
| Anti-CD31 | Rabbit | Abcam | ab281583 |
| Anti-α-SMA | Mouse | Abcam | ab7817 |
| Anti-α-SMA | Rabbit | Abcam | ab124964 |
| Anti-VE-cadherin | Rabbit | Abcam | ab33168 |
| Anti-Vimentin | Rabbit | Abcam | ab92547 |
| Anti-FSP1 | Rabbit | Abcam | ab197896 |
| Anti-GAPDH | Mouse | Beyotime | AG019-1 |
| Alexa Fluor 555 Donkey Anti-Mouse IgG |  | Beyotime | A0460 |
| Alexa Fluor 488 Goat Anti-Mouse IgG |  | Beyotime | A0428 |
| Alexa Fluor 488 Goat Anti-Rabbit IgG |  | Beyotime | A0423 |
| Alexa Fluor 555 Donkey Anti-Rabbit IgG |  | Beyotime | A0453 |
| HRP-labeled Goat Anti-Mouse IgG |  | Beyotime | A0216 |
| HRP-labeled Goat Anti-Rabbit IgG |  | Beyotime | A0208 |

**Table S2 Primer sequences for qRT-PCR**

| **Genes** | **Sequences** |
| --- | --- |
| mmu-Fn1 | F:5’-CTCTGCTCTTGGGGCTCAAC-3’  R:5’-GGAAAAGTCCTGAGGTGGGG-3’ |
| mmu-CTGF | F:5’-AGAACTGTGTACGGAGCGTG-3’  R:5’-GTGCACCATCTTTGGCAGTG-3’ |
| mmu-Col1a1 | F:5’-TAGGCCATTGTGTATGCAGC-3’  R:5’-ACATGTTCAGCTTTGTGGACC-3’ |
| mmu-Col3a1 | F:5’-CTGTAACATGGAAACTGGGGAAA-3’  R:5’-CCATAGCTGAACTGAAAACCACC-3’ |
| hsa-Fn1 | F:5’-CGGTGGCTGTCAGTCAAAG-3’  R:5’-AAACCTCGGCTTCCTCCATAA-3’ |
| hsa-CTGF | F:5’-CAGCATGGACGTTCGTCTG-3’  R:5’-AACCACGGTTTGGTCCTTGG-3’ |
| hsa-Col1a1 | F:5’-CACTTTCCACCCTCTCTCCA-3’  R:5’-AGGGGGAAAAACTGCTTTGT-3’ |
| hsa-Col3a1 | F:5’-GGAGCTGGCTACTTCTCGC-3’  R:5’-GGGAACATCCTCCTTCAACAG-3’ |
| mmu-α-SMA | F:5’-CAGGCATGGATGGCATCAATCAC-3’  R:5’-ACTCTAGCTGTGAAGTCAGTGTCG-3’ |
| mmu-CD31 | F:5’-ACGCTGGTGCTCTATGCAAG-3’  R:5’-TCAGTTGCTGCCCATTCATCA-3’ |
| mmu-VE-cadherin | F:5’-TCAACGCATCTGTGCCAGAGAT-3’  R:5’-CACGATTTGGTACAAGACAGTG-3’ |
| mmu-Vimentin | F:5’-CGTCCACACGCACCTACAG-3’  R:5’-GGGGGATGAGGAATAGAGGCT-3’ |
| hsa-α-SMA | F:5’-AAAAGACAGCTACGTGGGTGA-3’  R:5’-GCCATGTTCTATCGGGTACTTC-3’ |
| hsa-CD31 | F:5’-AACAGTGTTGACATGAAGAGCC-3’  R:5’-TGTAAAACAGCACGTCATCCTT-3’ |
| hsa-VE-cadherin | F:5’-CAGCCCAAAGTGTGTGAGAA-3’  R:5’-TGTGATGTTGGCCGTGTTAT-3’ |
| hsa-TCF12 | F:5’-AGTTATCCATCTCCTAAGCCACC-3’  R:5’-AAGAATTGTGGGTCCCATCTTG-3’ |
| hsa-Vimentin | F:5’-AGTCCACTGAGTACCGGAGAC-3’  R:5’-CATTTCACGCATCTGGCGTTC-3’ |
| GAPDH | F:5’-AAGAAGGTGGTGAAGCA-3’  R:5’-TCCACCACCCAGTTGCTGTA-3’ |
| hsa-TGF-β1 | F:5’-GGCCAGATCCTGTCCAAGC-3’  R:5’-GGGTTTCCACCATTAGCAC-3’ |

| **Genes** | **Sequences** |
| --- | --- |
| TGF-β1 | ChIP NC F:5'-ACGTGGCTAATTTTGTGGGTG-3' |
| TGF-β1 | ChIP NC R:5'-CTGAGGCGGGCTGATCACCTG-3' |
| TGF-β1 | ChIP1 F: 5'-GGCACGGGCTTTCGTGGGTG-3' |
| TGF-β1 | ChIP1 R: 5'-AGGCAGAGTCCCTCAGCACTCC-3' |
| TGF-β1 | ChIP2 F: 5'-GAGGCCCCCATGTTGACAGAC-3' |
| TGF-β1 | ChIP2 R: 5'-GCCAAGCGCCACCAAAGCGG-3' |

**Table S3 Primer sequences for ChIP**
