# Supplementary figures and images for: PD-1 inhibition disrupts collagen homeostasis and aggravates cardiac dysfunction through endothelial-fibroblast crosstalk and EndMT
Source: Front Pharmacol. 2025 Mar 17;16:1549487. doi: 10.3389/fphar.2025.1549487 (PMC11955664; doi:10.3389/fphar.2025.1549487)

Full Scan Images of Gels

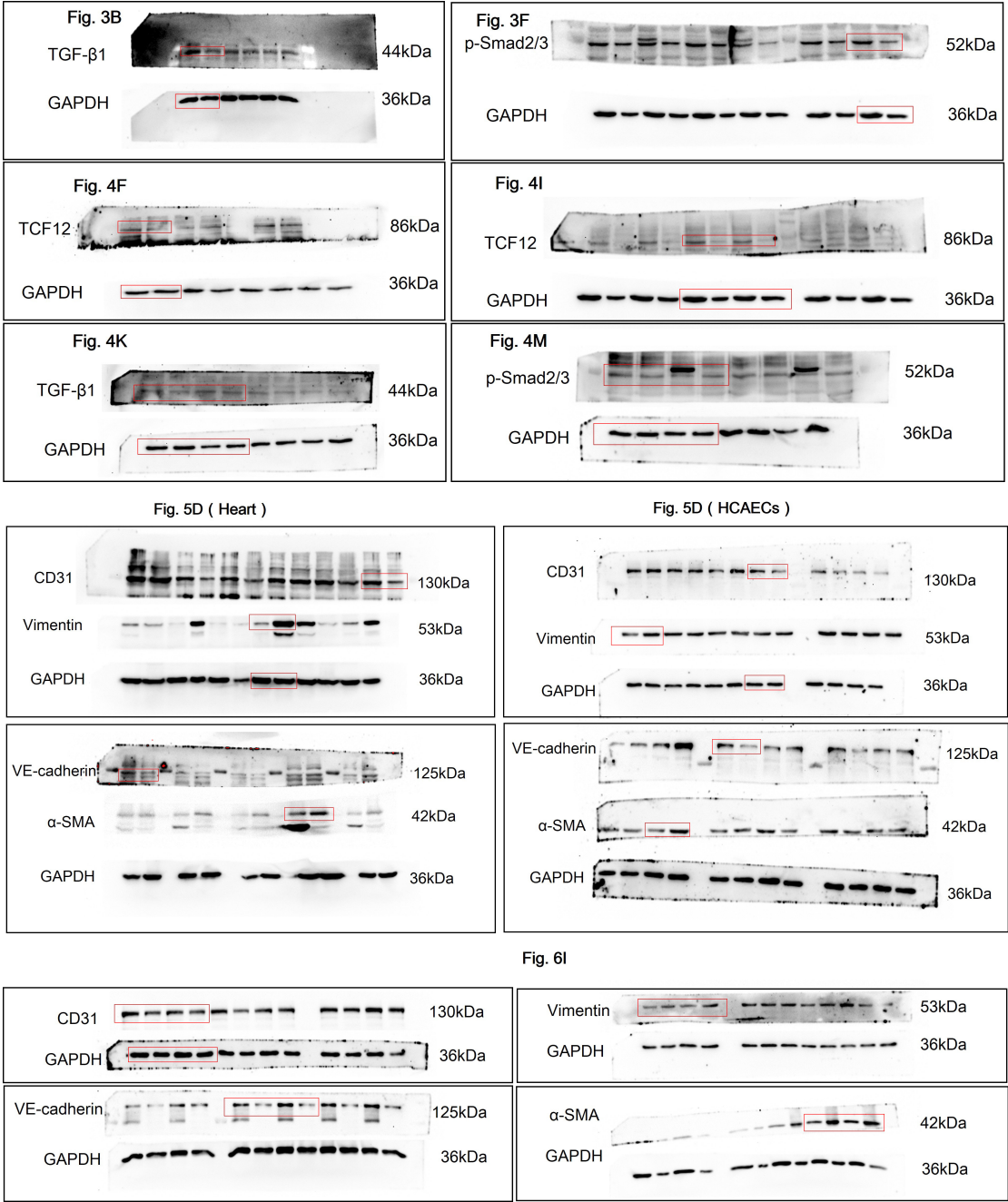

Supplement: Supplementary file 2 [file DataSheet1.PDF]
